# Effects of outdoor activity time, screen time, and family socioeconomic status on physical health of preschool children

**DOI:** 10.3389/fpubh.2024.1434936

**Published:** 2024-08-07

**Authors:** Bobo Zong, Lun Li, Yufang Cui, Wenxia Shi

**Affiliations:** ^1^College of Physical Education and Sports, Beijing Normal University, Beijing, China; ^2^School of P.E., China University of Geosciences, Wuhan, Hubei, China; ^3^China National Children’s Center, Beijing, China; ^4^Center Teacher Education Research, Beijing Normal University, Beijing, China

**Keywords:** outdoor activity time, screen time, family socioeconomic status (SES), physical health, preschool children

## Abstract

**Objective:**

Experienced 3 years of pandemic-induced home life, in the post-epidemic period, preschoolers in China are falling short of the World Health Organization’s standards for screen time and outdoor activities. This notably impacts their physical well-being. The study aims to probe the associations between screen time, outdoor activities, and the physical health of preschoolers, offering insights to shape interventions targeting myopia and obesity prevention in children.

**Methods:**

A cross-sectional study was conducted in Guangdong Province, involving a representative sample of 23,992 preschoolers and their caregivers recruited through proportional stratified cluster sampling. Data collection utilized the Chinese Early Human Capability Index (CHeHCI, eHCi), a questionnaire on children’s media use in daily family life, and Body Mass Index (BMI). Linear regression and binary logistic regression models were employed to analyze the impact of screen time and outdoor activity duration on the physical health of preschoolers.

**Results:**

In the high family socioeconomic status (SES) group, children had significantly less screen time compared to those in the medium and low SES groups. Outdoor activity time varied significantly based on SES, with higher SES linked to extended outdoor engagement. Additionally, children’s eHCi health dimension score exhibited significant SES-related differences, showcasing higher scores for children in higher SES groups. In terms of gender differences, boys dedicated significantly more time to outdoor activities than girls, yet boys had a notably higher overweight rate. Furthermore, girls demonstrated better health outcomes based on eHCi health scores. A significant association emerged between overweight and screen time in children with high SES, indicating that prolonged screen time was linked to a higher likelihood of overweight based on BMI. Additionally, a substantial negative correlation was observed between children’s eHCi health dimension score and screen time. Furthermore, children’s outdoor activity time exhibited a significant positive correlation with eHCi health dimension score. Regression analysis revealed that screen time could significantly negatively predict children’s physical health score, while outdoor activity time could significantly positively predict children’s eHCi physical health score.

**Conclusion:**

The current study highlights that family SES, age, and gender play pivotal roles in influencing preschoolers’ screen time and outdoor activity duration, with family SES being particularly influential. Higher family SES correlates with reduced screen time, increased outdoor activity, and elevated health levels among children. Importantly, children’s screen time negatively predicts their health status, while outdoor time positively predicts their health status.

## 1 Introduction

Health behaviors established during the ages of 3–6 constitute pivotal determinants influencing the subsequent well-being of children. In recent years, health issues prevalent among children, including conditions like obesity and myopia, exhibit a concerning trend characterized by increased incidence at younger ages (1, 2). These conditions exert a substantial impact on the physical and mental health of children, prompting heightened societal attention (3, 4). Research findings underscore that augmented screen time and diminished engagement in physical activity emerge as critical factors contributing to prevalent health issues among children, encompassing concerns such as overweight, obesity, and myopia (5, 6).

Screen time refers to the duration during which individuals focus their attention on electronic devices with screens (7). Meta-analyses have revealed that excessive screen time increases the risk of overweight or obesity in children, with a daily screen time exceeding 2 h exacerbating this risk (8, 9). Furthermore, prolonged screen time may result in the deprivation of sleep, a reduction in physical activity, and subsequent adverse effects on physical health (10). The health standards established by the World Health Organization (WHO) for physical activity and sedentary behavior in children under 5 are explicit. Children aged 1–2 are recommended to engage in a minimum of 3 h of daily physical activity, with screen time not exceeding 1 h. Between the ages of 3 and 5, the recommendation is to participate in at least 1 h of moderate to high-intensity physical activity per day, with a screen time not exceeding 1 h (11, 12). Additionally, the American Academy of Pediatrics underscores the importance of limiting screen time to no more than 2 h per day for children above 2 years old, while those under 2 years old are discouraged from any screen exposure (13).

Surveys conducted in various regions reveal concerning trends, indicating that more than half of young children excessively use screens. Moreover, domestic scholars have conducted surveys and studies on the physical activity levels of 3–5-year-old children in Nanchang, Nanjing, and Beijing. Findings indicate that the compliance rate for model to vigorous physical activity (MVPA) for 3–6-year-old children in Jiangxi ranges from 45 to 61% (14). In Jiangsu, the average MVPA duration for 3–6-year-old is 58 min, with a compliance rate of about 43% (15). Notably, the compliance rate for MVPA in five kindergartens in Beijing exceeds 90% (16). This underscores an unfavorable compliance status regarding healthy behaviors such as screen time and physical activity among young children in China, marked by significant regional variations. Further analysis suggests that these disparities may be attributed to differences in family SES (17, 18).

Family SES exerts a significant influence on the overall well-being of preschool children (19, 20). Those hailing from families with higher SES are more likely to access resources encompassing education, recreation, and entertainment (21–23). This increased access affords them opportunities to engage with a diverse array of stimuli and participate in outdoor activities, consequently contributing to a reduction in screen time among children. Existing research suggests that outdoor activities, as integral components of physical activity, positively correlate with children’s physical health (24). The greater the duration of outdoor activity, the higher the index of children’s physical health (9). Despite these insights, it remains uncertain whether variations exist in screen time and outdoor activities among preschool children across diverse SES, genders, and age groups within different family contexts. In addition, it is still unclear how different family socioeconomic levels affect the BMI and eHCi of preschool children of different ages and genders. Considering that excessive screen time and less time outdoors may have an impact on preschoolers’ physical health, while numerous studies have explored the interplay between family social status, physical activity, and screen time among preschool children (25–27), the specific relationships among outdoor activity time, screen time, and the overall physical health of preschoolers under distinct family social statuses remain unclear.

Building upon this foundation, the study initially comprehensively assessed the family’s social status, screen time, outdoor activity duration, and the fundamental health status of preschool children. Then, it conducted an analysis to discern the influence of varying family SES, gender, and age among preschool children on screen time, outdoor activities and physical health. Compared with previous studies, based on the physical health of preschool children, this study analyzed in more detail the gender differences and age differences in outdoor time, screen time, BMI and physical health index eHCi of preschool children under different family socioeconomic levels. When measuring the physical health status of children, in addition to using the commonly used BMI in previous studies, this study exploratory used the health dimension in the Chinese Early Human Capability Index (eHCI), making the exploration of children’s physical health more comprehensive and in-depth. Finally, the investigation delved into unraveling the intricate relationship between outdoor activity duration, screen time, and the physical health of preschool children within diverse family social statuses.

The underlying assumption posits potential disparities in the impact of screen time and outdoor activities among preschool children contingent on differing SES, genders, and age groups across various family settings. Furthermore, the hypothesis anticipates that preschool children hailing from low SES families may exhibit elevated screen time coupled with diminished outdoor activity duration, consequently manifesting weaker physical health in comparison to their counterparts from families with medium to high SES. In contrast, school-age children within the middle to high socioeconomic strata are anticipated to display reduced screen time, augmented outdoor activity duration, and consequently, enhanced physical health compared to their peers with lower SES.

## 2 Materials and methods

### 2.1 Study design

This study is a cross-sectional study, using survey data from the 20182019 Guangdong Kindergarten Children’s Educational Experience and Family Life Survey Project led by the research team of Guangdong Preschool Education Teacher Training Center and Beijing Normal University. The core team of this project brings together multiple researchers across fields, institutions, and methodologies. It aims to explore the educational experience and family life status of preschool children in China from a broader perspective. This survey covers kindergartens in 12 cities in Guangdong Province, including the west coast of the Pearl River Delta, the Pearl River Delta, the central area of the Pearl River Delta, the east wing, the west wing, and the mountainous area. The reason why Guangdong Province is chosen for the study is mainly because Guangdong Province is located in the southern coastal area of China and has a wide area. According to Qipu data, the province’s population ranks first in China and the population composition is complex, with many local indigenous people, while the total size of the migrant population is as high as 29.62 million, accounting for 23.5% of the total population, ranking first in the country, and the floating population is more. In addition, there is a large difference in the economy of the province, and even a pattern of “one province and three worlds.” From the perspective of *per capita* GDP, the *per capita* GDP of Shenzhen, the “richest,” is 29,900 US dollars, which is more than seven times that of Meizhou in the same province (3,996 US dollars). This also laid a certain foundation for the analysis of SES divided into three groups: high, middle, and low.

Then stratified sampling was carried out according to ownership and quality level of kindergartens. About 10–20 kindergartens were selected from each city, and three classes were selected from each kindergarten (one for each primary school and one for each major school). All teachers and principals of the sampled kindergartens were enrolled, and all parents of the sampled classes were enrolled.

In addition, in order to improve the representativeness of the sample, the research team tried to meet the following sampling requirements: (1) Economic development level of the park: covering different districts, counties, communities, or streets with good, medium, and poor local economic development level; (2) Urban and rural distribution of parks: at least one township central park or rural kindergarten should be selected from each place; and (3) Kindergarten size: under the same conditions, priority will be given to kindergartens with six or more classes.

### 2.2 Participants

According to the stratified sampling principle, parents of each child were contacted by teachers of the sampled class teachers and asked to fill out an electronic questionnaire. Because this questionnaire is based on the survey of the overall situation of the family, only one person in a family can fill out the questionnaire. This questionnaire is mainly presented in the form of electronic questionnaire, which is sent by the teacher of the sample class to the parents of each child, and the parents are asked to send the screenshot to the teacher after the answer is finished, so as to ensure that every sample family must fill in the questionnaire to the greatest extent.

Due to the wide coverage and large sample size, parents who answered the questionnaire could not be interviewed at the school, so the project team explained and trained the principal of the sample, and then the principal trained the teachers of the sample class, and finally the teachers explained the parents in detail. All data are anonymized to ensure the privacy of each parent and child. There is a paragraph of information before the questionnaire, which is regarded as informed consent, without a separate signed informed consent. Data were collected from 26,621 kindergarten children and their parents, equal proportion stratified cluster sampling was used in this study. After deleting invalid questionnaires and eliminating outliers according to box plot, 23,992 valid questionnaires were obtained, and the effective questionnaire recovery rate was 90.12%. Among them, there are a total of 12,835 males and 11,157 females. There are a total of 2,983 children aged 3; 7,782 children aged 4; 7,586 children aged 5, and 5,110 children aged 6.

### 2.3 Measurements

#### 2.3.1 Family questionnaire on children’s multimedia usage

In the data collection process, we utilized the Family Questionnaire on Children’s Multimedia Usage, specifically designed to investigate screen time among kindergarten children. This questionnaire was adapted from the British Children’s Multimedia Use Survey, the American Children’s Multimedia Use Survey for ages 0–8, and a survey on children’s multimedia use in Beijing conducted by Li and Wang (28). Comprising four distinct aspects, this study primarily focused on the section related to “children’s multimedia use at home,” encompassing a total of four questions and 33 items. The pivotal query, “Children’s daily family life allocation,” was employed to gauge children’s daily screen time and outdoor activity time. The survey employed an eight-point scoring method, with 1 indicating 0 min and 8 denoting 2 h or more. The internal consistency of this scale was robust, with the main variable was 0.79 in our study.

#### 2.3.2 Children’s ability index scale

We assessed the physical health of kindergarten children using the Early Human Capacity Index, with the Chinese version developed and adapted by the Shanghai Children’s Medical Center and China Development Research Foundation under the guidance of Sally Brinkman (29). This scale, featuring nine dimensions and 60 questions, was primarily focused on the physical health dimension in this study, incorporating four items such as “Whether the child often gets sick” and “Whether the child has a disability or special needs.”

These specific items served as indicators of physical health in our survey, and responses were recorded using a “Yes/no” binary scoring method, where 1 indicated “Yes” and 0 indicated “no.” Prior analyses indicated that a higher mean of all questions correlated with a higher level of development in children’s health dimensions. However, it is noteworthy that, based on the reliability and validity test of the Chinese version of the tool, the reliability coefficient for the subscale related to the physical health dimension was relatively low (*α* = 0.18) (30). In our study, the reliability coefficient for this dimension is 0.26. Considering that the child’s body mass index is a crucial indicator for assessing their health, this dimension, serving as the outcome variable, we will be combined with the child’s body mass index to further explore the physical health status of children.

#### 2.3.3 Children’s body mass index

Body mass index of a child is calculated by dividing their weight by the square of their height (kg/m^2^), with the child’s height (cm) and weight (kg) reported by a parent or caregiver. In this study, the physical health status of children was evaluated based on the Body Mass Index standard for children aged 3–6, as outlined by the World Health Organization (WHO), to ascertain whether they fell within the overweight category. This criterion, endorsed by the World Health Organization, has been consistently applied in previous research to gauge the health status of preschool children (31, 32).

#### 2.3.4 Family socioeconomic status

Drawing on the findings from prior research, which underscored associations between family SES and children’s screen time, this study incorporates family SES as a control variable. The creation of the Family SES Index adhered to the methodologies employed by Ren (33) in conjunction with the technical report of the International Student Assessment Project PISA 2009 (34). Employing principal component analysis, factors such as education level, annual family income, and occupational class were identified to ensure consistency. Utilizing the formula SES = (0.58 * highest education level + 0.59 * highest occupational class +0.55 * monthly average family income)/0.63, the corresponding socio-economic status value for the child’s family was computed. A higher SES value indicates a higher social status for the child’s family. Because the questionnaire focuses on the family as a whole rather than the respondent’s own situation, the study measures the age of the parents, the number of children in the family, and the family structure in terms of demographic variables. Detailed data are shown in Table 1.

**Table 1:** Age of parents, family structure, and number of children in the family.

	Parental age (year)		Family structure		Number of children (*n*)
	Father	Mother	Nuclear family	Extended family	
Low-SES	34.81	32.36	2,376	3,450	2.03
Middle-SES	35.38	32.99	4,677	7,219	1.77
High-SES	36.97	34.49	2,456	3,814	1.67
Total	35.66	33.23	9,509	14,483	1.8

### 2.4 Statistical analysis

Stata 15.0 software was employed for the normal analysis and variance homogeneity test of all data. The collected data underwent several analyses. Descriptive analyses were utilized to examine the SES, screen time, outdoor activity time, and health of preschool children in Guangdong Province. To explore the similarities and differences in outdoor activity time, screen time, and physical health among kindergarten children based on varying family SES, age, and gender, ANOVA and Chi-square tests were conducted. Levene’s test was applied to assess the equality of variances across groups, followed by the Bonferroni’s *post hoc* test. Correlation analysis was employed to discern the relationship between outdoor activity time, screen time, and physical health. Additionally, linear regression and binary logistic regression analyses were carried out to delve into the predictive outcomes of screen time and outdoor activity time on children’s physical health.

## 3 Results

### 3.1 Feature analysis of SES, screen time, time spent outdoors, and child health in preschool children

In this study, preschool children were classified into three groups based on their SES values: the low SES group (SES values lower than the lower quartile; *N* = 5,826), the high SES group (SES values greater than the higher quartile; *N* = 6,270), and the middle SES group (SES values between the lower quartile and the higher quartile; *N* = 11,896). Detailed data regarding screen time, outdoor activity time, and child health for preschoolers in these SES groups are presented in Tables 2, 3.

**Table 2:** The screen time, time spent outdoors, and eHCi health dimensions in children with different family SES ( $\bar{x}$ ±s).

	3-year old		4-year old		5-year old		6-year old		Total		
Low-SES group											
Number	Girl (*n* = 395)	Boy (*n* = 427)	Girl (*n* = 863)	Boy (*n* = 1,044)	Girl (*n* = 827)	Boy (*n* = 926)	Girl (*n* = 537)	Boy (*n* = 640)	Girl (*n* = 2,703)	Boy (*n* = 3,123)	Total (*N* = 5,826)
Time											
Screen time (min)	105.09 ± 82.44	105.74 ± 87.01	105.39 ± 90.78	115.05 ± 95.18	113.06 ± 93.38	114.2 ± 93.51	115.53 ± 95.82	120.22 ± 99.38	109.73 ± 91.43	114.96 ± 95.80	112.53 ± 93.82
Outdoor activity time (min)	40.68 ± 35.46	46.67 ± 39.28	45.95 ± 36.49	47.8 ± 38.97	45.13 ± 38.43	49.75 ± 39.09	48.81 ± 39.08	52.15 ± 39.08	45.61 ± 37.65	48.96 ± 39.03	47.40 ± 38.43
Health											
eHCi health dimension (score)	0.85 ± 0.19	0.84 ± 0.19	0.86 ± 0.18	0.84 ± 0.19	0.88 ± 0.18	0.88 ± 0.18	0.91 ± 0.17	0.88 ± 0.18	0.87 ± 0.18	0.86 ± 0.19	0.86 ± 0.18
Middle-SES group											
Number	Girl (*n* = 710)	Boy (*n* = 778)	Girl (*n* = 1,793)	Boy (*n* = 2,103)	Girl (*n* = 1,767)	Boy (*n* = 2,003)	Girl (*n* = 1,090)	Boy (*n* = 1,379)	Girl (*n* = 5,484)	Boy (*n* = 6,412)	Total (*N* = 11,896)
Time											
Screen time (min)	106.04 ± 88.3	107.29 ± 89.09	108.16 ± 94.40	109.42 ± 86.79	116.01 ± 100.68	114.5 ± 92.31	116.92 ± 96.84	118.57 ± 101.36	112.5 ± 96.81	112.91 ± 92.53	112.72 ± 94.52
Outdoor activity time (min)	53.13 ± 38.35	53.85 ± 38.48	52.47 ± 39.96	56.57 ± 39.88	54.96 ± 39.74	58.69 ± 40.78	55 ± 40.04	57.85 ± 39.66	53.82 ± 39.66	57.15 ± 39.99	55.62 ± 39.87
Health											
eHCi health dimension (score)	0.86 ± 0.18	0.85 ± 0.19	0.88 ± 0.18	0.87 ± 0.18	0.89 ± 0.18	0.88 ± 0.18	0.9 ± 0.17	0.90 ± 0.16	0.89 ± 0.18	0.88 ± 0.18	0.88 ± 0.18
High-SES group											
Number	Girl (*n* = 324)	Boy (*n* = 349)	Girl (*n* = 941)	Boy (*n* = 1,038)	Girl (*n* = 990)	Boy (*n* = 1,073)	Girl (*n* = 675)	Boy (*n* = 789)	Girl (*n* = 2,970)	Boy (*n* = 3,300)	Total (*N* = 6,270)
Time											
Screen time (min)	100.23 ± 91.30	96.22 ± 84.78	99.04 ± 82.15	97.81 ± 92.60	98.16 ± 82.96	105.17 ± 94.45	101.31 ± 90.91	100.7 ± 83.03	99.6 ± 85.71	100.76 ± 90.40	100.21 ± 88.20
Outdoor activity time (min)	59.52 ± 40.63	60.19 ± 39.35	60.68 ± 40.29	63.41 ± 40.66	59.32 ± 41.52	64.82 ± 41.38	57.44 ± 38.39	64.34 ± 40.45	59.32 ± 40.33	63.54 ± 40.63	61.54 ± 40.54
Health											
eHCi health dimension (score)	0.88 ± 0.18	0.86 ± 0.18	0.89 ± 0.17	0.88 ± 0.18	0.90 ± 0.16	0.91 ± 0.16	0.91 ± 0.17	0.91 ± 0.17	0.9 ± 0.17	0.89 ± 0.17	0.89 ± 0.17

eHCi, The early human capacity index; F, Female; M, Male.

**Table 3:** The overweight and normal-weight body mass index of children in the different family SES [*N*(%)].

	3-year old		4-year old		5-year old		6-year old		Total		
Low-SES group											
Number	Girl (*n* = 327)	Boy (*n* = 365)	Girl (*n* = 702)	Boy (*n* = 860)	Girl (*n* = 679)	Boy (*n* = 776)	Girl (*n* = 460)	Boy (*n* = 533)	Girl (*n* = 2,232)	Boy (*n* = 2,601)	Total (*n* = 4,833)
Overweight	18 (5.50)	32 (8.77)	35 (4.99)	87 (10.12)	44 (6.48)	83 (10.70)	21 (4.57)	45 (8.44)	118 (5.29)	247 (9.50)	365 (7.55)
Normal	309 (94.50)	333 (91.23)	667 (95.01)	773 (89.88)	635 (93.52)	693 (89.30)	439 (95.43)	488 (91.56)	2,114 (94.71)	2,354 (90.50)	4,468 (92.45)
Middle-SES group											
Number	Girl (*n* = 594)	Boy (*n* = 640)	Girl (*n* = 1,458)	Boy (*n* = 1,753)	Girl (*n* = 1,471)	Boy (*n* = 1,631)	Girl (*n* = 916)	Boy (*n* = 1,182)	Girl (*n* = 4,543)	Boy (*n* = 5,327)	Total (*n* = 9,870)
Overweight	34 (5.72)	48 (7.50)	70 (4.80)	116 (6.62)	46 (3.13)	103 (6.32)	18 (1.97)	71 (6.01)	168 (3.70)	338 (6.35)	506 (5.13)
Normal	560 (94.28)	592 (92.50)	1,388 (95.20)	1,637 (93.38)	1,425 (96.87)	1,528 (93.68)	898 (98.03)	1,111 (93.99)	4,375 (96.30)	4,989 (93.65)	9,364 (94.87)
High-SES group											
Number	Girl (*n* = 276)	Boy (*n* = 296)	Girl (*n* = 782)	Boy (*n* = 856)	Girl (*n* = 822)	Boy (*n* = 898)	Girl (*n* = 577)	Boy (*n* = 660)	Girl (*n* = 2,489)	Boy (*n* = 2,752)	Total (*n* = 5,241)
Overweight	18 (6.52)	11 (3.72)	29 (3.71)	49 (5.72)	25 (3.04)	50 (5.57)	13 (2.25)	47 (7.12)	85 (3.42)	157 (5.70)	242 (4.62)
Normal	258 (93.48)	285 (96.28)	753 (96.29)	807 (94.28)	797 (96.96)	848 (94.43)	564 (97.75)	613 (92.88)	2,404 (96.58)	2,595 (94.30)	4,999 (95.38)

For the assessment of children’s body mass index, as per previous studies, it was categorized into two dimensions: non-overweight and overweight. It is noteworthy that, due to anomalies in the weight data, the sample size for children’s screen time, outdoor time, health dimension score of eHCi, and family SES was 23,992 children, while the sample size for body mass index was 19,944 children.

The data revealed that the daily screen time for all children was 109.41 ± 92.9 min. The screen time for children in the middle and low SES groups was comparable (middle SES groups vs. low SES groups: 112.72 ± 94.52 vs. 112.53 ± 93.82), respectively, with the screen time for children in the high SES group being the lowest (100.21 ± 88.2 min). Overall, 15,761 (65.69 percent) children exceeded the 1-h screen time recommendation by the American Association of Pediatrics. Among them, 8,052 (67.69%) children in the middle SES group and 3,900 (66.94%) children in the low SES group had more than 1 h of screen time, while the minimum proportion of 3,809 (60.75%) children in the high SES group exceeded 1 h of screen time.

### 3.2 Differences in screen time, outdoor activity time, and child health among SES groups, ages, and genders

#### 3.2.1 SES difference

In this study, disparities in screen time, outdoor time, body mass index, and health dimension scores of eHCi among children in various SES groups were investigated. Screen time, outdoor activity time, and eHCi health dimension scores served as observational variables, with the SES group of children employed as the independent variable for ANOVA are presented in Tables 2, 3.

Our findings revealed a significant reduction in the screen time of children in the high SES group compared to those in the middle and low SES groups (*p* < 0.001). Furthermore, children’s outdoor activity time exhibited a notable difference across SES groups (*p* < 0.001), with higher SES associated with prolonged outdoor activity time. Additionally, the health dimension score of children’s eHCi also displayed significant variations based on SES (*p* < 0.001), indicating that higher SES correlated with elevated health dimension scores in children’s eHCi. As body mass index was dichotomized into overweight and non-overweight variables in this study, the Chi-square test was utilized to explore the SES group differences in body mass index. The results underscored a significant correlation between children’s overweight status and SES groups (*p* < 0.001).

#### 3.2.2 Gender differences

To examine gender disparities in children’s screen time, outdoor activity time, body mass index, and health dimension scores of eHCi, this study treated screen time, outdoor activity time, and health dimension score of eHCi as distinct observational variables. Children’s gender was considered the independent variable, and independent sample T-test analyses were conducted.

As shown in Table 4, the results revealed no significant gender-based differences in screen time among the three SES groups (*p* > 0.05). Concerning outdoor activity time, a statistically significant difference emerged between boys and girls (*t* = 6.77, *p* < 0.001), with boys spending more time outdoors than girls. This gender disparity remained statistically significant both overall and within SES groups. Notably, the proportion of boys classified as overweight (7.47%) exceeded that of girls (4.17%).

**Table 4:** Differences in screen time, outdoor activity time, and eHCi health dimensions among children in three SES groups.

	Low-SES group	Middle-SES group	High-SES group
Time			
Screen time (min)	2.12	0.24	0.52
Outdoor activity time (min)	3.32^③^	4.55^③^	4.12^③^
Health			
eHCi health dimension (score)	−2.63^②^	−1.85^①^	−0.86

^①^*p* < 0.05, ^②^*p* < 0.01, ^③^*p* < 0.001.

Regarding the eHCi health dimension score, statistically significant differences between boys and girls were observed overall and within the middle and low SES groups. Girls exhibited higher eHCi health dimension scores than boys. However, no significant gender difference in the eHCi health dimension score was noted among children in the high SES group (*p* > 0.05).

#### 3.2.3 Age difference

To assess age-related disparities in children’s screen time, outdoor activity duration, body mass index, and eHCi health dimension scores, ANOVA was conducted are presented in Tables 2, 3. The findings revealed that, concerning screen time, there was an overall increase with the age of children (*p* < 0.05). Notably, children aged 6 years in the low SES group exhibited significantly higher screen time than their 3-year-old counterparts (*p* < 0.05). Conversely, for children in the high SES group, there was no statistically significant difference between screen time and age (*p* > 0.05).

Regarding outdoor activity time, there was a notable overall increase for children aged 4–6 compared to 3-year-old (*p* < 0.01). In the middle SES group, the outdoor activity time of 5-year-old was significantly higher than that of 3-year-old (*p* < 0.05). Nevertheless, for children in the high SES group, there was no statistically significant difference in outdoor activity time and age (*p* > 0.05).

Concerning eHCi health dimension scores, an overall rise was observed with the age of children (*p* < 0.001). In the low and high SES groups, except for the 3–4 and 5–6 age groups, children’s eHCi health dimension scores exhibited a significant age-related difference (*p* < 0.001). The older the age, the higher the score of children’s eHCi health dimension.

Chi-square testing indicated no significant difference between children with low SES and high SES (*p* > 0.05). However, in the middle SES group, the proportion of overweight children gradually decreased with the increase of children’s age, dropping from 7.12% at 3 years old to 4.43% at 6 years old.

### 3.3 Correlation analysis of screen time, outdoor activity time, and children’s health

To examine the interrelationships between screen time, outdoor activity duration, body mass index, and eHCi health dimension, correlation analysis was employed (refer to Table 5). The outcomes of the Pearson correlation method indicated a significant correlation between overweight and screen time in both the overall children and those in the high SES group (*p* < 0.001). This implies that a lengthier screen time was associated with a higher likelihood of children being overweight. However, there was no significant correlation between BMI and screen time in the middle and low SES groups. A noteworthy negative correlation (*p* < 0.001) emerged between children’s eHCi health scores and screen time, whether considered collectively or stratified into low, middle, and high SES groups. This signifies that an extended screen time was linked to lower eHCi health scores in children. In both the overall and low SES groups, children’s screen time exhibited a significant positive correlation with age, though no significant correlation was observed in the high SES group. Furthermore, both overall and within specific groups, a significant positive correlation (*p* < 0.001) was identified between children’s screen time and outdoor activity duration.

**Table 5:** Relationship of screen time, outdoor activity time, and physical health of children in different family SES.

	Screen time	Outdoor activity time	BMI	eHCi health dimension	Age	Gender
Total						
Screen time	1					
Outdoor activity time	0.29^③^	1				
BMI	0.02^③^	−0.01	1			
eHCi health dimension	−0.1^③^	0.06^③^	0	1		
Age	0.03^③^	0.03^③^	−0.02^②^	0.08^③^	1	
Gender	−0.01	−0.04^③^	−0.06^③^	0.02^②^	−0.01	1
Low-SES group						
Screen time	1					
Outdoor activity time	0.35^③^	1				
BMI	0.02	−0.01	1			
eHCi health dimension	−0.08^③^	0.06^③^	−0.01	1		
Age	0.04^②^	0.05^③^	0	0.10^③^	1	
Gender	−0.03^①^	−0.04^③^	−0.08^③^	0.03^②^	0	1
Middle-SES group						
Screen time	1					
Outdoor activity time	0.29^③^	1				
BMI	0.01	0	1			
eHCi health dimension	−0.1^③^	0.05^③^	0	1		
Age	0.04^③^	0.03^②^	−0.04^②^	0.08^③^	1	
Gender	0	−0.04^③^	−0.06^③^	0.02	−0.02	1
High-SES group						
Screen time	1					
Outdoor activity time	0.26^③^	1				
BMI	0.05^③^	−0.01	1			
eHCi health dimension	−0.1^③^	0.05^③^	0.01	1		
Age	0.01	0	0	0.08^③^	1	
Gender	−0.01	−0.05^③^	−0.05^③^	0.01	−0.01	1

BMI, Body mass index; eHCi, The early human capacity index; ^①^*p* < 0.05, ^②^*p* < 0.01; ^③^*p* < 0.001.

Regarding outdoor activity time, both collectively and when stratified by groups, there was no significant correlation between children’s outdoor activity time and BMI (*p* > 0.05). However, a noteworthy positive correlation emerged between outdoor activity time and children’s eHCi health dimension scores (*p* < 0.001). A positive correlation between children’s age and outdoor activity time was observed in both the overall dataset and the middle and low SES groups. In this study, boys were assigned a value of 0, and girls were assigned a value of 1. The data demonstrated a significant negative correlation (*p* < 0.001) between gender, outdoor activity time, and BMI in both the overall dataset and the three SES groups. Furthermore, a significant positive correlation (*p* < 0.001) between age and children’s eHCi health dimension scores was evident both overall and across the three SES groups.

### 3.4 Regression analysis of screen time, outdoor time, and children’s health

In order to explore the distinct predictive impacts of screen time and outdoor activity time on children’s physical health, regression analysis was conducted while controlling for children’s gender and age. Screen time and outdoor activity time were considered as independent variables, with eHCi physical health dimension score and body mass index designated as dependent variables.

#### 3.4.1 The early human capacity index health dimension

The eHCi health dimension score, being a continuous variable, underwent linear regression analysis after adjusting for children’s age and gender (see Table 6). The results indicate that both screen time and outdoor activity time significantly predict the scores of children’s eHCi physical health dimensions, whether considering the overall children or the three groups of high, medium, and low SES. In this context, screen time emerges as a significant negative predictor of children’s eHCi physical health scores (*p* < 0.001), while outdoor activity time stands out as a significant positive predictor of these scores (*p* < 0.001). The explanatory rate for outdoor activity time and high SES group children’s screen time is 1%, whereas the explanatory rate of screen time in medium-low SES group children and overall children’s screen time is 2%.

**Table 6:** Coefficients from linear regression models estimating eHCi health dimension.

	Screen time			Outdoor activity time		
	*ß*	*R^2^*	*F*	*ß*	*R^2^*	*F*
Total eHCi health dimension	−0.1^③^	0.02	140.9	0.06^③^	0.01	84.28
Low-SES eHCi health dimension	−0.09^③^	0.02	34.45	0.06^③^	0.01	25.84
Middle-SES eHCi health dimension	−0.1^③^	0.02	69.42	0.05^③^	0.01	32.8
High-SES eHCi health dimension	−0.1^③^	0.01	31.8	0.05^③^	0.01	17.73

SES, Social economic status; eHCi, The Early Human Capacity Index; ^①^*p* < 0.05, ^②^*p* < 0.01, ^③^*p* < 0.001; the age and sex were set control variables.

#### 3.4.2 Body mass index

As previously mentioned, this study categorized body mass index into two dimensions: non-overweight and overweight, employing binary logistic regression for analysis. Model 1 and Model 2, respectively, depict the influence of overall children’s screen time and outdoor activity time on children’s body mass index. Model 3 and Model 4, respectively, illustrate the impacts of screen time and outdoor activity time on children’s body mass index in the low SES group. Model 5 and Model 6, respectively, demonstrate the effects of screen time and outdoor activity time on children’s body mass index in the middle SES group; while Model 7 and Model 8, respectively, portray the effects of screen time and outdoor activity time on children’s body mass index in the high SES group (see Table 7).

**Table 7:** Binary logistic regression analysis affecting body mass index in children.

Factors		Screen time	Outdoor activity time	Age	Gender
Model 1	*ß*	0.1^③^		−0.1^②^	−0.58^③^
	*OR*	1.1		0.91	0.56
	*95%CL*	0.04 ~ 0.15		−0.16 ~ −0.04	−0.71 ~ −0.46
Model 2	*ß*		−0.04	−0.09^②^	−0.59^③^
	*OR*		0.96	0.91	0.56
	*95%CL*		−0.11 ~ 0.02	−0.16 ~ −0.03	−0.72 ~ −0.46
Model 3	*ß*	0.08		−0.02	−0.63^③^
	*OR*	1.08		0.98	0.53
	*95%CL*	−0.02 ~ 0.18		−0.13 ~ 0.09	−0.85 ~ −0.40
Model 4	*ß*		−0.03	−0.01	−0.63^③^
	*OR*		0.97	0.99	0.53
	*95%CL*		−0.15 ~ 0.08	−0.12 ~ 0.1	−0.86 ~ −0.40
Model 5	*ß*	0.05		−0.17^③^	−0.57^③^
	*OR*	1.05		0.84	0.56
	*95%CL*	−0.04 ~ 0.13		−0.27 ~ −0.08	−0.76 ~ −0.39
Model 6	*ß*		0	−0.17^③^	−0.57^③^
	*OR*		1	0.84	0.56
	*95%CL*		−0.09 ~ 0.09	−0.26 ~ −0.08	−0.76 ~ −0.38
Model 7	*ß*	0.19^③^		−0.02	−0.53^③^
	*OR*	1.21		0.98	0.59
	*95%CL*	1.09 ~ 1.34		−0.16 ~ 0.11	−0.8 ~ −0.26
Model 8	*ß*		−0.04	−0.02	−0.55^③^
	*OR*		0.96	0.98	0.58
	*95%CL*		−1.73 ~ 0.08	−0.15 ~ 0.12	−0.82 ~ −0.28

^①^*p* < 0.05, ^②^*p* < 0.01, ^③^*p* < 0.001.

The specific results are as follows: there is a significant relationship (*p* < 0.001) between screen time and BMI overweight in all children and children in the high SES group. The longer the screen time, the greater the risk of BMI overweight in children. There was no significant relationship between outdoor activity time and children’s BMI (*p* > 0.05). A significant correlation exists between gender and BMI in children (*p* < 0.001), with boys having a higher risk of being overweight than girls. For overall children and children in the middle SES group, there is a significant relationship between age and BMI, with younger children at a higher risk of developing BMI as overweight compared to older children.

## 4 Discussion

In recent years, the surge in childhood obesity, myopia, and other health issues has become a growing concern, largely attributed to the rapid development of social economy and technology (2, 35). Study has identified the imbalance between screen time and outdoor activities as a primary factor contributing to these health challenges in children (36, 37). Additionally, variations in family SES may exert a certain degree of influence on children’s screen usage and outdoor activity durations, thereby impacting their overall health (38, 39). This study aims to evaluate the impacts of outdoor activity time, screen time, and family SES on the physical health of preschool children in the contemporary era.

### 4.1 Factors influencing screen time, time spent outdoors in preschoolers

This study reveals that the screen time and outdoor activity duration of our 3–6-year-old preschoolers fall short of the recommendations set by the American Academy of Pediatrics (40, 41). Extensive screen time and limited outdoor activities may result in prolonged sedentary behavior, leading to various physiological issues such as visual impairment, overweight, obesity, and compromised sleep quality. Furthermore, these habits may adversely affect language development and cognitive function, posing a threat to overall physical health (41–43). Our analysis of the correlation between screen time, outdoor activity duration, and health-related indicators in school-age children supports these findings. Specifically, we observed a significant negative correlation between children’s eHCi health dimension score and screen time, while outdoor activity time exhibited a significant positive correlation with eHCi health dimension scores.

Hence, we speculate that the surge in obesity and myopia rates among preschool children in China could be linked to prolonged screen time and inadequate outdoor activities. Numerous factors, including family background and personal characteristics, influence individuals’ screen usage and outdoor activity participation. Given the unique nature of preschool children, this study primarily explores the intrinsic and extrinsic factors impacting them.

#### 4.1.1 Immanent cause

Our study suggests that gender and age (44, 45) may serve as crucial internal factors influencing preschoolers’ screen time, outdoor activity duration, and health. Surprisingly, we discovered no statistically significant difference between boys and girls concerning screen time and outdoor activity duration. However, girls exhibited better eHCi scores and BMI than boys. Although boys spent significantly more time outdoors, they scored lower than girls in the physical health dimension.

Based on the developmental patterns of young children, we speculate the higher rate of overweight in boys compared to girls may result from a combination of factors, including body structure, morphology, and energy metabolism in both genders (46). Moreover, boys’ more active and mischievous nature might lead to increased outdoor activities compared to girls. Our correlation analysis, treating gender as a categorical variable (0 for boys, 1 for girls), confirmed a significant negative correlation between gender, outdoor activity duration, and BMI, providing partial validation for our rationale.

Age is another potential factor influencing the screen time, outdoor time, and physical fitness of preschoolers (47). As discussed earlier, older preschoolers tend to engage in more activities, leading to longer activity durations. Research indicates that as preschool children age, their social cognition and interests increase, resulting in higher screen time (48, 49). Our findings align with this trend, particularly among preschool children with significant age differences, while no significant differences were observed among those with smaller age gaps. Notably, older age correlates with higher health dimension scores and a lower incidence of overweight.

#### 4.1.2 External cause

Research has consistently highlighted the significance of family social status as an influential external factor impacting preschoolers’ screen time, outdoor activity duration, and overall physical health (50). Notably, lower screen time was associated with higher family social status, while outdoor activity time displayed significant variability across SES levels (51). Employing correlation analysis, we examined the interplay between screen time, outdoor activities, and the physical health of preschoolers from diverse family social statuses. The outcomes revealed a significant positive correlation between outdoor activity time and children’s eHCi health dimension scores. Conversely, screen time exhibited a significant negative correlation with children’s eHCi health dimension scores. These findings underscored that higher SES levels were linked to longer outdoor activity durations and reduced screen time, thereby validating our initial hypothesis. Subsequent analysis of physical health dimensions and BMI in preschool children across different family social statuses consistently supported this trend: higher family social status corresponded to elevated eHCi scores and a lower incidence of overweight status.

Family socio-economic status appears to set off a chain of interconnected reactions: varying socio-economic statuses among families lead to divergent behaviors and perspectives among parents (52). These parental disparities, in turn, exert an influence on the screen time and outdoor activity durations of their children (53). Notably, our study observed that children in high SES groups exhibited the lowest screen time across different family socio-economic statuses. This suggests that these families possess ample financial resources, enabling their children to explore a broader array of experiences. Moreover, parents in high socio-economic status families, owing to their elevated education levels and professional experiences, may harbor a heightened awareness of their children’s health and education (54). In contrast, parents with lower economic and social statuses are often preoccupied with work and domestic responsibilities, leaving them with limited time and energy to dedicate to their children. Consequently, these parents may opt for screen-based activities such as games, mobile phone videos, or television to occupy their children’s time. The ongoing pandemic has further exacerbated this situation, with home-based online learning contributing to increased screen time and decreased outdoor activity time (55, 56). Additionally, the surge in short-form videos, coupled with technological advancements, has led platforms to algorithmically recommend more content to children, intensifying their reliance on screens and elongating screen time (57).

### 4.2 Effects of screen time and outdoor activity on physical health in preschoolers

To delve deeper into the predictive capacity of screen time and outdoor activity time on children’s physical health, we employed linear regression analysis. Controlling for gender and age, with family SES as a grouping variable, screen time and outdoor activity time were designated as independent variables, and eHCi physical health dimension scores and body mass index served as dependent variables. Our study findings revealed that both screen time and outdoor activity time significantly predicted the physical health scores of children’s eHCi, encompassing the overall children and the three groups stratified by SES. Specifically, screen time emerged as a significant negative predictor of the physical health scores of children’s eHCi (*p* < 0.001), signifying that a reduction in screen time corresponds to an enhancement in the physical health of preschoolers. Conversely, outdoor activity time emerged as a significant positive predictor of the physical health scores of children’s eHCi (*p* < 0.001), indicating that increased outdoor activity time aligns with improved health outcomes among preschoolers. Consequently, it can be inferred that the physical health of preschoolers exhibits predictability based on their screen time and outdoor activity time (58, 59).

Simultaneously, recognizing BMI as another pivotal indicator for assessing the physical health of preschool children (60), we employed binary logistic regression to scrutinize the influence of screen time and outdoor activity time on the body mass index of preschoolers. The outcomes revealed no significant association between outdoor activity time and the likelihood of children being overweight. However, a notable correlation was observed between screen time and the BMI of preschool children. Increased screen time corresponded to a heightened risk of children having an overweight BMI. This substantiates the substantial impact of screen time on the physical health of preschool children (61). Consequently, to a certain extent, the physical health of preschoolers can be prognosticated based on their screen time and outdoor activity time (62, 63). Building upon our findings, we fervently recommend that families undertake their supervisory roles, with parents leading by example to curtail screen time. Instead, they should explore alternative outdoor activities that foster children’s concentration and reduce their reliance on screens, especially in families with lower social status.

### 4.3 Practical implications

This study boasts several strengths; foremost among them is the inclusion of physical health indices, specifically children’s eHCi health dimension score and BMI. This is noteworthy as these indices have not been extensively addressed in the majority of prior studies that examined outdoor activity time, screen time, and family SES in preschool children. To our knowledge, this investigation represents the inaugural exploration of the effects of screen time and outdoor activities on the physical health of preschool children in the post-epidemic era. Finally, a significant contribution of this study is the establishment of a predictive link between screen time, outdoor activity time, and the physical health of preschoolers.

### 4.4 Study limitation and future research

While our study offers valuable insights into the interplay of screen time, outdoor activity, and family SES on the physical health of preschool children, it is crucial to acknowledge certain limitations. The focus on preschoolers aged 3–6 necessitated reliance on parental responses to questionnaires, potentially introducing biases and discrepancies between reported data and actual behaviors. This reliance on subjective reporting may impact the precision and reliability of our findings.

Additionally, our study is geographically constrained to regions with higher economic development levels. The exclusion of economically underdeveloped areas limits the generalizability of our results. Future research endeavors should aim for a more diverse sample, including areas with varying economic statuses, to enhance the external validity and applicability of our conclusions.

Moreover, this study is based on the “Guangdong Kindergarten Children’s Educational Experience and Family Life Survey Project,” so the data used in this study are all children who have received preschool education. This is indeed a limitation of this study, and future studies should supplement the data for this subset of children as much as possible.

In conclusion, recognizing these limitations is essential for a nuanced interpretation of our study findings. Future research should address these constraints to provide a more comprehensive understanding of the complex relationships among screen time, outdoor activities, and the physical health of preschool children across diverse socio-economic contexts.

## 5 Conclusion

Impacted by the 3-year epidemic, the screen time and outdoor activity levels of preschool children in China during the post-epidemic period fall below the standards recommended by the World Health Organization, significantly impacting their physical well-being. Our findings underscore the influential role of family SES, age, and gender in shaping the screen time and outdoor activity patterns of preschoolers, with family SES emerging as a particularly significant factor.

Our results reveal a discernible correlation between family SES and children’s screen time, indicating that families with higher SES tend to limit their children’s screen time. Additionally, a positive association is observed between family SES and outdoor activity time, suggesting that higher SES is linked to increased time spent outdoors. Notably, these trends extend to overall health outcomes, with higher family SES correlating with enhanced health levels. Furthermore, our analysis indicates that children’s screen time serves as a negative predictor of their health status, while outdoor time acts as a positive predictor. These findings emphasize the intricate interplay between socioeconomic factors and lifestyle choices, underscoring the need for targeted interventions and educational initiatives to address disparities and enhance the physical health of preschoolers in the post-epidemic era in China. It is necessary for families, schools, and communities to work together in the future to analyze the physical health level of preschool children of different genders and ages under different family socioeconomic levels, and take some measures or interventions to reduce the screen time of preschool children. Increase their outdoor activity time, thereby reducing the incidence of overweight and obesity, in order to enhance the physical health of preschool children.

## Data availability statement

The original contributions presented in the study are included in the article/supplementary material; further inquiries can be directed to the corresponding author.

## Ethics statement

This study was conducted based on approval from the Beijing Normal University and schools that participated in the survey. The studies were conducted in accordance with the local legislation and institutional requirements. Written informed consent for participation was not required from the participants or the participants’ legal guardians/next of kin in accordance with the national legislation and institutional requirements. The participant’s legal guardian/next of kin was informed about the study at the start of the questionnaire and consented to the participant’s participation in the study when filling out the questionnaire.

## Author contributions

BZ: Writing – review & editing, Writing – original draft, Resources, Methodology, Conceptualization. LL: Writing – original draft, Investigation. YC: Writing – original draft, Investigation. WS: Writing – review & editing, Writing – original draft, Methodology, Conceptualization.
